# Correction: Simião et al. Involvement of Inflammatory Cytokines, Renal NaPi-IIa Cotransporter, and TRAIL Induced-Apoptosis in Experimental Malaria-Associated Acute Kidney Injury. *Pathogens* 2024, *13*, 376

**DOI:** 10.3390/pathogens14101058

**Published:** 2025-10-20

**Authors:** Gustavo Martins Simião, Kleber Simônio Parreira, Sandra Gabriela Klein, Flávia Batista Ferreira, Fernanda de Souza Freitas, Eduardo Ferreira da Silva, Neide Maria Silva, Murilo Vieira da Silva, Wânia Rezende Lima

**Affiliations:** 1 Faculty of Health Sciences, Federal University of Rondonopolis, Rondonópolis 78736-900, MT, Brazil; gustavo.simiao@aluno.ufr.edu.br; 2 Institute of Biotechnology, Federal University of Catalao, Catalão 75706-881, GO, Brazil; kleberparreira@ufcat.edu.br (K.S.P.); fernandade@discente.ufcat.edu.br (F.d.S.F.); eduardo_silva@discente.ufg.br (E.F.d.S.); 3 Laboratory of Biotechnology in Experimental Models, Federal University of Uberlandia, Uberlândia 38410-337, MG, Brazil; klein.sandra@ufu.br (S.G.K.); flaviabatistaf@ufu.br (F.B.F.); murilo.vieira@ufu.br (M.V.d.S.); 4 Institute of Biomedical Sciences, Federal University of Uberlandia, Uberlândia 38405-318, MG, Brazil; nmsilva@ufu.br

## Missing Supplemental Materials

In the original publication [1], the Supplementary Materials file was not included. The authors state that the scientific conclusions are unaffected. This correction was approved by the Academic Editor. The original publication has also been updated.
