# Supplementary material for: Involvement of Inflammatory Cytokines, Renal NaPi-IIa Cotransporter, and TRAIL Induced-Apoptosis in Experimental Malaria-Associated Acute Kidney Injury
Source: Pathogens. 2024 May 1;13(5):376. doi: 10.3390/pathogens13050376 (PMC12557330; doi:10.3390/pathogens13050376)
Supplement: Supplementary file 1 [file pathogens-13-00376-s001.zip › pathogens-2890661-supplementary.pdf]

Table S1. Primers for real-time RT-PCR.

Primer	Forward (5′-3′)	Reverse (5′-3′)
NaPi-IIa	CATCACAGAGCCCTTCACAA	CGTTTGGGGTCTGTCTCAAT
IL-1β	TGACAGTGATGAGAATGACCTGTTC	TTGGAAGCAGCCCTTCATCT
IL-18	CTGCCTGCATCACACAAAGA	GCAGAGCAGCTGGAATTCTC
IFN-γ	GGCTGTTTCTGGCTGTTACTGC	CATCCTTTTGCCAGTTCCTCC
TNF-α	CCACCACGCTCTTCTGTCTACTG	GGCTTGTCACTCGAATTTTGAGA
TRAIL	CCCTGCTTGCAGGTTAAGAG	GGCCTAAGGTCTTTCCATCC
CASP3	GGGCCTGTTGAACTGAAAAA	AGCCTCCACCGGTATCTTCT
CASP8	CCCCAAATGTAAGCTGGAAG	AGCAGGCTCTTGTTGACCTG
BCL-2	AGTACCTGAACCGGCATCTG	CTCTGAAGACCCTGCTCAGC
GAPDH	TGCACCACCAACTGCTTAGC	GGATGCAGGGATGATGTTCT

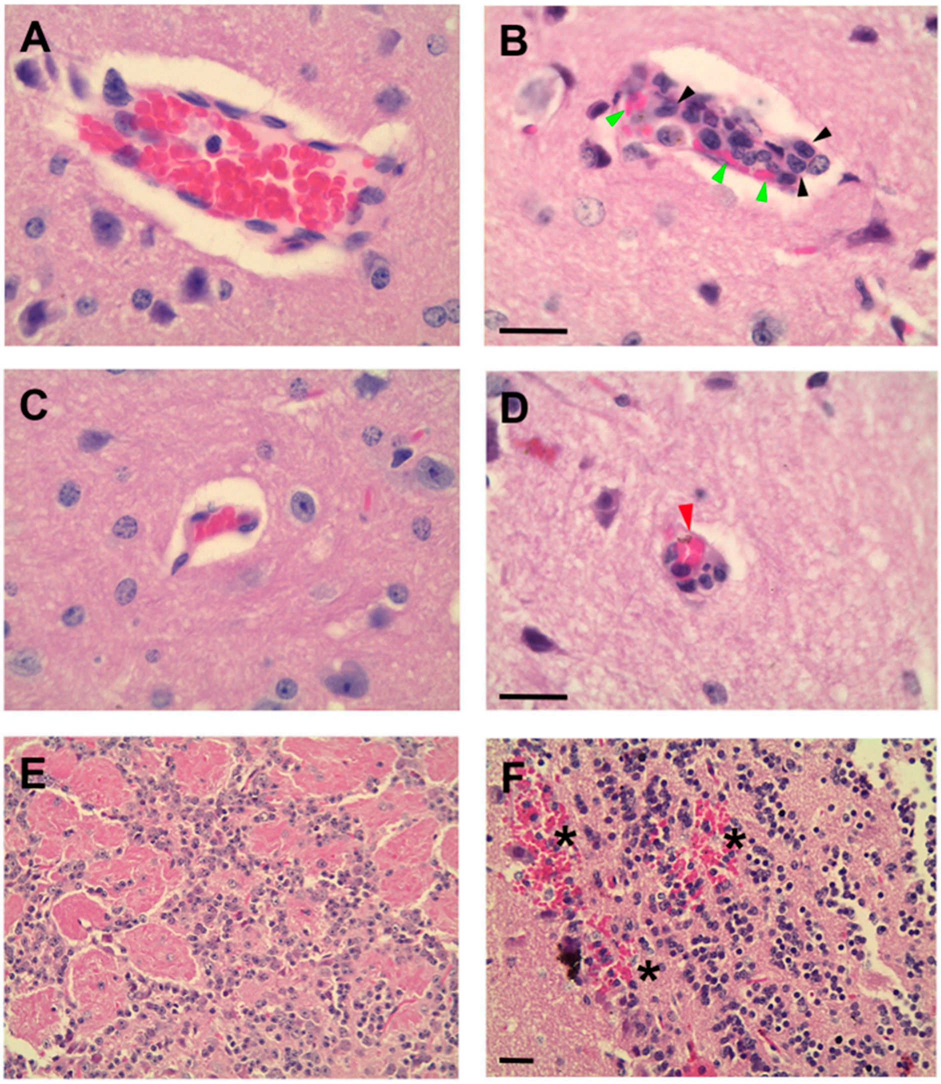

Figure S1. Histopathological analysis of brain tissue sections. (**A**, **C**, **E**) HE-stained brain sections from uninfected control mice. All panels from uninfected mice exhibit absence of histopathological changes. (**B**, **D**, **F**) HE-stained brain sections from PbA-infected animals. (**B**) HE-stained brain tissue that presents migrating leukocytes (black arrow heads) and sequestered iRBCs (green arrow heads) in a blood vessel. (**D**) HE-stained brain section that shows a blood vessel with leukocytes and a rosette-like formation (red arrow head) containing iRBCs. (**F**) HE-stained brain section that reveals microhemorrhage foci in regions from the olfactory bulb (asterisks). These brain tissue changes correspond to some of the common histopathological features that characterize ECM. Tissue samples were harvested from mice euthanized at day 7 post-infection. **A** – **D**, original magnification: x100; **E**, **F**: x40. Scale: 40 μm

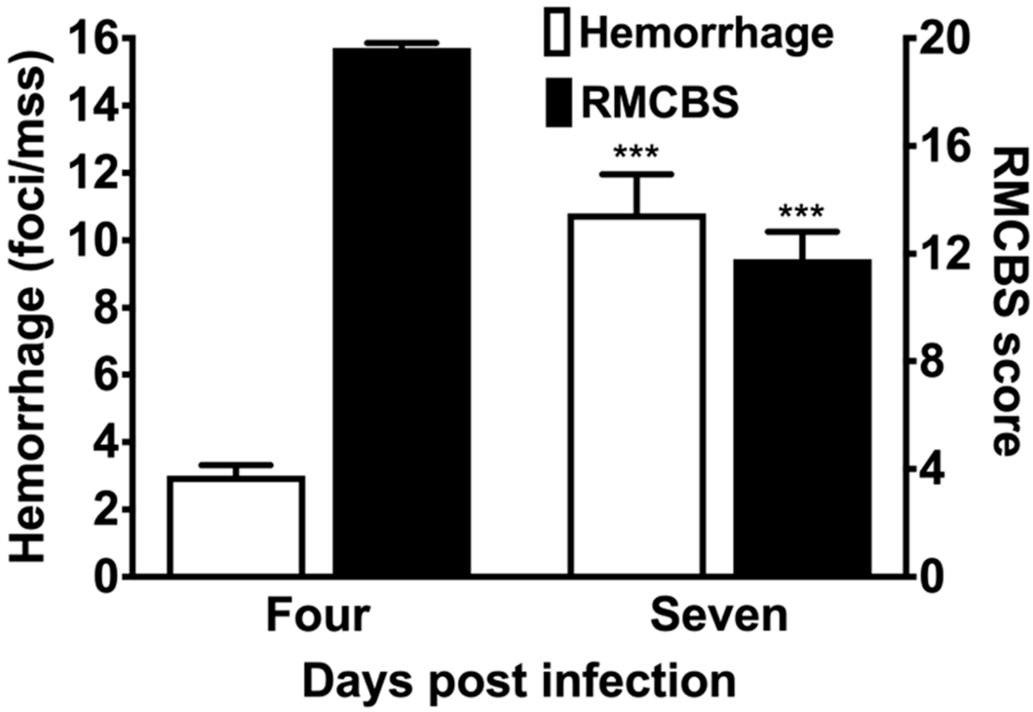

Figure S2. Comparative analysis of RMCBS scores and the incidence of brain microhemorrhages at 4 and 7 days post-infection. Quantitative examination of microhemorrhage foci in the total area of HE-stained midsagittal sections (mss) revealed that the mean number of foci is about three-fold higher in mice at day 7 (10.8 ± 1.16 foci/mss; mean ± SEM; n = 5; *** $p = 0.000$) compared with animals at day 4 (3.00 ± 0.32 foci/mss; n = 5) after PbA infection. By contrast, the mean RMCBS score was significantly lower for mice with 7 days (11.8 ± 1.02; mean ± SEM; n = 5; *** $p = 0.000$) of infection compared with mice with 4 days (19.63 ± 0.18; n = 5), indicating that cognitive and motor functions may not decline until 6 or 7 days of PbA infection when the number of microhemorrhage foci in the brain tissue is substantially high to promote a reduction in RMCBS scores to approximately 12. Tissue samples were harvested from mice euthanized at days 4 and 7 post-infection.

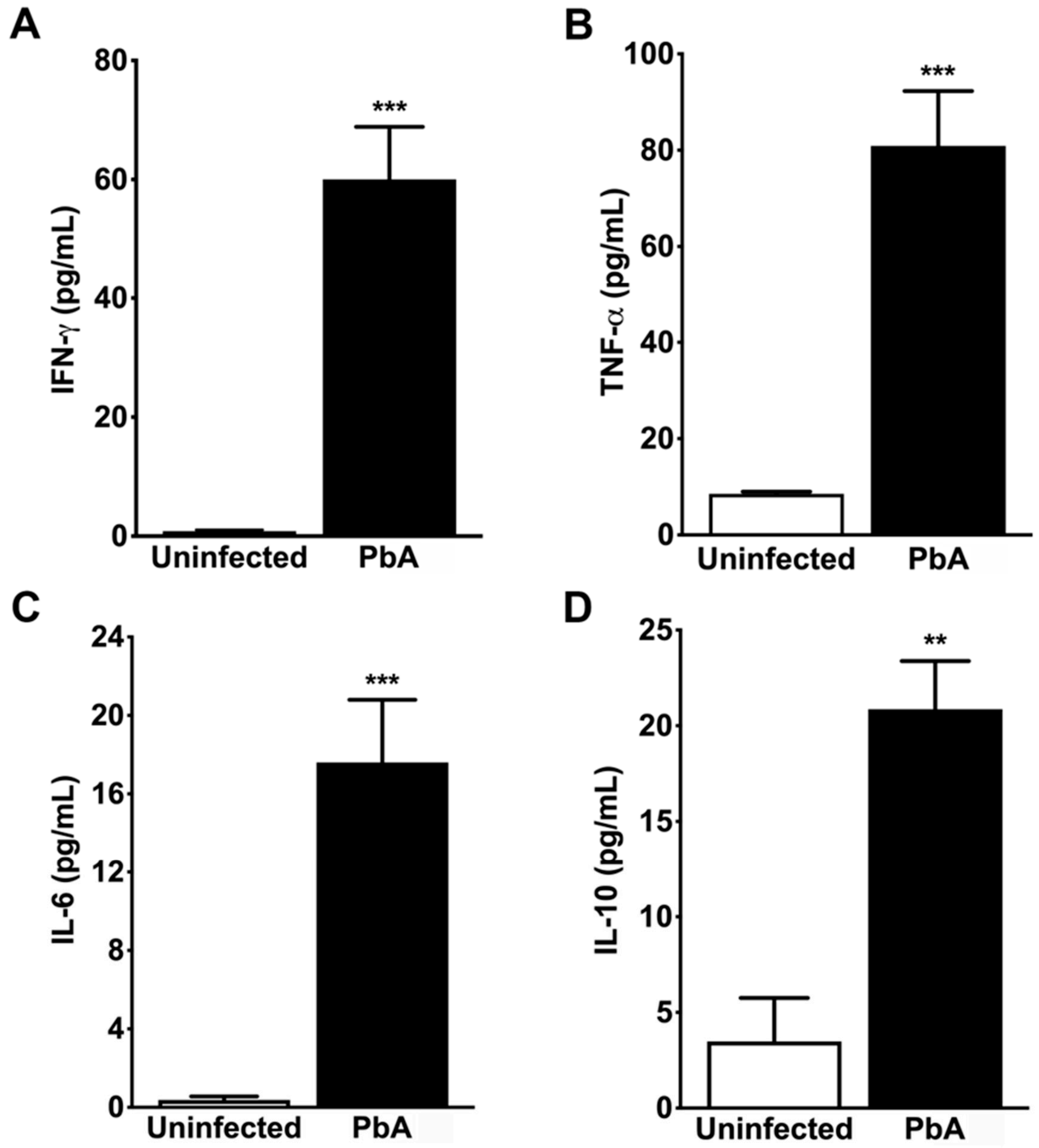

Figure S3. CBA analysis of cytokines in serum. (**A**) Cytokine measurement shows a very strong induction of IFN-γ in serum samples from PbA-infected mice (49.32 ± 11.24 mg/dL; ***p = 0.006) as compared with uninfected controls (0.83 ± 0.23 mg/dL) after 7 days of inoculation. (**B**) Cytokine measurements also show that TNF-α concentration in the serum was approximately 10 times higher in infected mice (80.88 ± 11.39 mg/dL; ***p = 0.000) than in uninfected controls (8.54 ± 0.43 pg/mL). (**C**) CBA analysis reveals that PbA infection potently enhances IL-6 production in serum of infected mice (17.61 ± 3.19 mg/dL; ***p = 0.000) in contrast with uninfected controls (0.37 ± 0.18 pg/mL). (**D**) Cytokine measurement shows significantly higher IL-10 levels in serum from PbA-infected mice (20.86 ± 2.52 mg/dL; **p = 0.001) than in control animals (3.49 ± 2.27 mg/dL). This potent elevation in the serum levels of IL-6, IFN-γ, and TNF-α cytokines, as part of *Plasmodium*-induced inflammation, may likely strengthen a "cytokine storm", resulting in multiple organ failures, which include the brain and kidney. N = 5 and n=7 for uninfected controls and PbA-infected mice, respectively. The RMCBS scores for these animals were 19.00 ± 0.32 and 11.57 ± 0.30, respectively. All animals were euthanized on day 7 post-infection, and serum samples were collected.

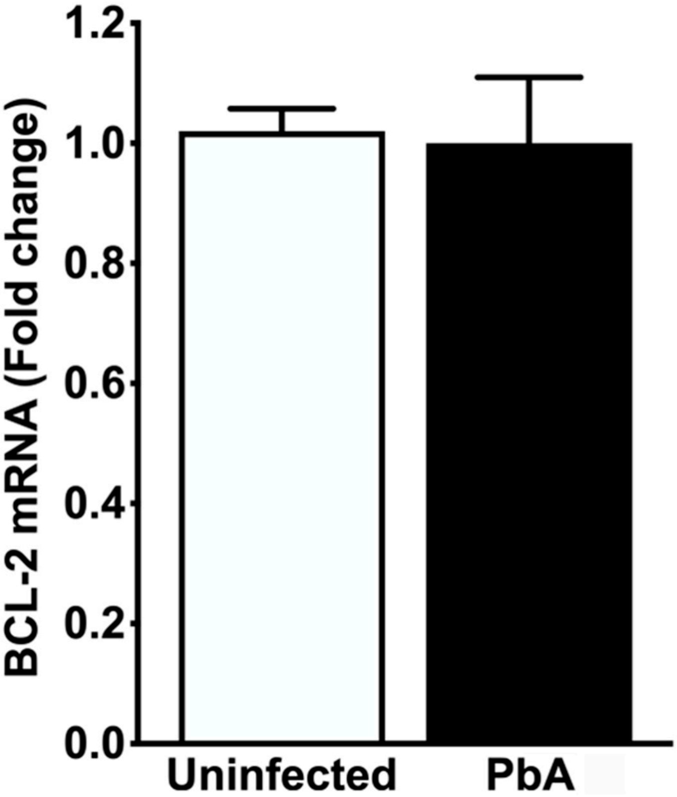

Figure S4. Gene expression of BCL-2 in the whole kidney. Quantitative RT-PCR analysis shows that BCL-2 mRNA levels in PbA-infected mice (1.00 ± 0.11 fold change; $p = 0.867$) was not significantly different of that of uninfected controls (1.02 ± 0.04 fold change). These results suggest that the intrinsic apoptosis pathway may not be stimulated in kidney cells of mice with ECM. N = 5 for uninfected controls and PbA-infected mice. The RMCBS scores for these animals were 19.41 ± 0.25 and 11.60 ± 0.25, respectively. All animals were euthanized on day 7 post-infection, and tissue samples were harvested.
